# Fibroblasts – the cellular choreographers of wound healing

**DOI:** 10.3389/fimmu.2023.1233800

**Published:** 2023-08-14

**Authors:** Samuel Knoedler, Sonja Broichhausen, Ruiji Guo, Ruoxuan Dai, Leonard Knoedler, Martin Kauke-Navarro, Fortunay Diatta, Bohdan Pomahac, Hans-Guenther Machens, Dongsheng Jiang, Yuval Rinkevich

**Affiliations:** ^1^ Department of Plastic Surgery and Hand Surgery, Klinikum Rechts der Isar, Technical University of Munich, Munich, Germany; ^2^ Division of Plastic Surgery, Department of Surgery, Yale School of Medicine, New Haven, CT, United States; ^3^ Division of Plastic Surgery, Department of Surgery, Brigham and Women’s Hospital, Harvard Medical School, Boston, MA, United States; ^4^ Institute of Regenerative Biology and Medicine, Helmholtz Zentrum München, Munich, Germany; ^5^ Department of Hand, Plastic and Reconstructive Surgery, Microsurgery, Burn Trauma Center, BG Trauma Center Ludwigshafen, University of Heidelberg, Ludwigshafen, Germany

**Keywords:** fibroblast, fibroblast diversity, wound healing, wound repair, skin injury, fascia, skin regeneration

## Abstract

Injuries to our skin trigger a cascade of spatially- and temporally-synchronized healing processes. During such endogenous wound repair, the role of fibroblasts is multifaceted, ranging from the activation and recruitment of innate immune cells through the synthesis and deposition of scar tissue to the conveyor belt-like transport of fascial connective tissue into wounds. A comprehensive understanding of fibroblast diversity and versatility in the healing machinery may help to decipher wound pathologies whilst laying the foundation for novel treatment modalities. In this review, we portray the diversity of fibroblasts and delineate their unique wound healing functions. In addition, we discuss future directions through a clinical-translational lens.

## 1 Introduction

The fascial system is composed of continuous three-dimensional viscoelastic sheets of connective tissue matrix that envelopes and interpenetrate and connects between organs, muscles, and nerves (1). The traditional concept of fascia as a mere passive structure in force transmission is gradually becoming outdated. Recent research findings have identified the fascia as a three-layered mechano-metabolic framework, with central roles during skin development, wound repair, and scarring. Importantly, scar tissue formation and skin contraction are achieved by the active transport of extracellular matrix (ECM) from the fascia, deep beneath the wound edges (2, 3). This unidirectional transport of ECM is mediated by the collective migration of a fibrogenic cell lineage termed Engrailed-1-lineage positive fibroblasts (EPFs) (4). Accordingly, fascia transport by fascial fibroblasts is a key step in wound closure and scar formation. Genetic depletion of fascial fibroblasts halts fascia transport into wounds and results in chronic non-healing wounds (2, 5). Yet, throughout the process of wound healing, fibroblasts play a multifaceted role that extends beyond fascia mobilization and patch-like repair. In light of this broad remit, fibroblasts are heterogeneous in embryonic origin, plasticity, and functionality (6, 7).

As the body’s integumentary system, the skin is vulnerable to injury and trauma. In order to restore dermal and epidermal integrity in a timely manner, wound healing proceeds in a highly coordinated and complex cascade that can be subdivided into three sequential yet overlapping stages: Inflammation, proliferation, and remodeling (8). Throughout this temporally- and spatially-harmonized interplay of different cell types, fibroblasts carry a pivotal role in all stages of wound healing (9, 10) As such, their field of activity is broad, ranging from the deposition of ECM components through wound contraction to inflammation and scarring (11). Within their broad operational capacity, fibroblasts are able to differentiate from progenitor cells with a characteristic spindle-shaped appearance to postmitotic polygonal fibrocytes and myofibroblasts (12, 13). These subsets of fibroblasts have been shown to release different cytokines and exert varying effects during wound repair (4, 14–17). While this phenotype-adaptability of fibroblasts is well-researched, its implications for basic science and ramifications in clinical care are yet to be determined (18). Therefore, this review aims to discuss the heterogeneity of connective tissue fibroblasts, with a particular focus on wound healing and its translational prospects. A comprehensive understanding of fibroblasts’ diversity may help decipher wound pathologies and, ultimately, pave the way toward establishing new treatment modalities. Consequently, we shed light on future perspectives through a translational lens, bridging the gap between bench and bedside.

## 2 Heterogeneity and diversity of skin fibroblasts

**Figure 1:**
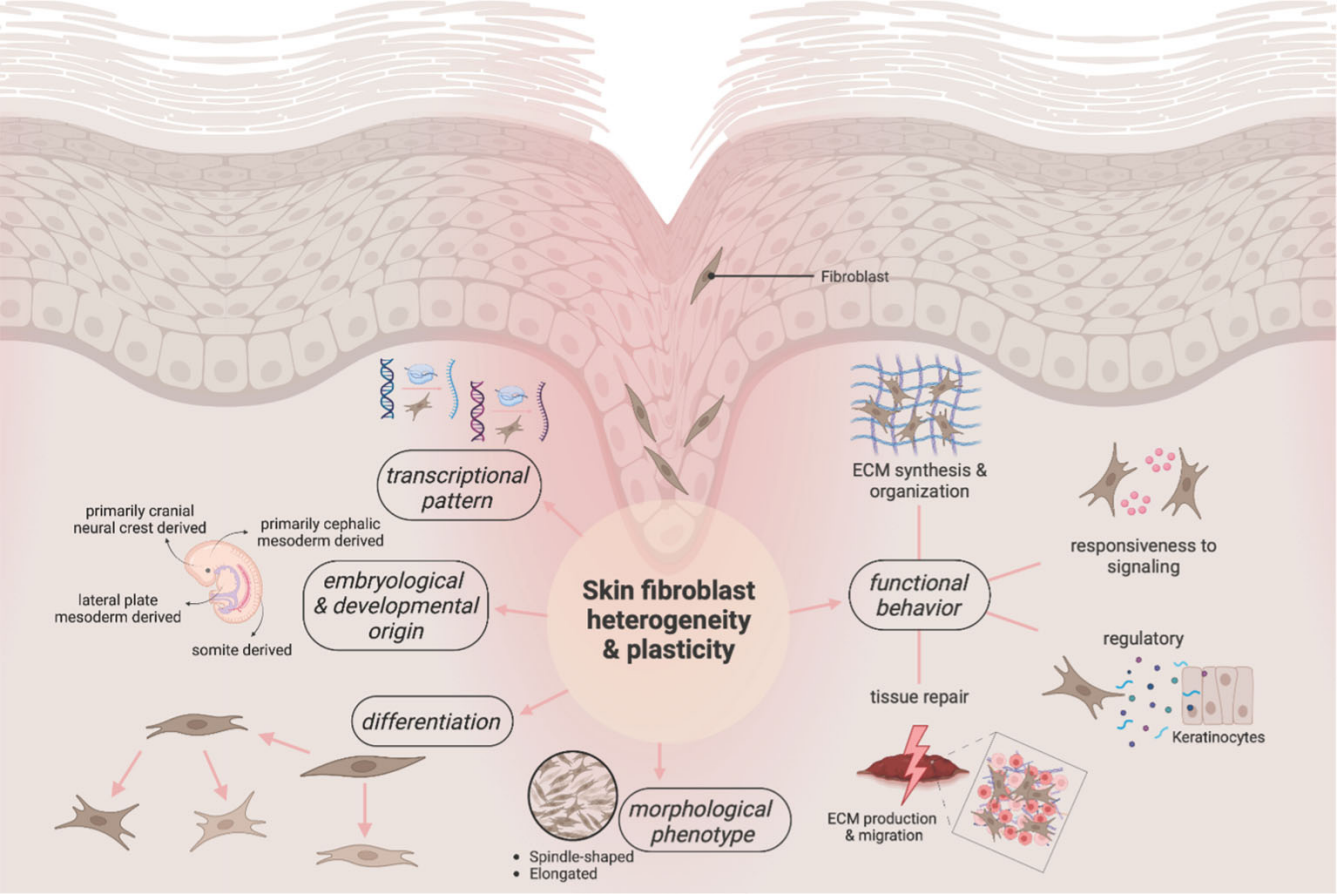
Overview of the multi-dimensional heterogeneity and plasticity of fibroblasts. Long misunderstood as inert components of the skin, the current line of evidence portrays fibroblasts as diverse and versatile cells. As early as during development, the first pillar of heterogeneity is introduced into this cell population, with different embryonic origins potentially determining the eventual postnatal functionality. Their heterogeneity is further reflected in a unique adaptability and (trans)differentiability. These chameleon-like capacities translate into heterogenous phenotypic markers and transcriptomic profiles of fibroblasts. As a result, “fibroblast” is a broad umbrella term that – depending on the lens through which it is studied – conveys a varying picture of these heterogenous cells.

Historically, connective tissues have been viewed as inert with homogeneous fibroblasts. Yet, this concept has gradually been replaced by a revised understanding which takes their multi-dimensional heterogeneity into account (**Figure 1**) (19, 20). In fact, these stromal cells show high variance in gene expression and function both between organ systems and within a single tissue (21, 22). In essence, fibroblasts’ diversity is largely due to differences in gene expression patterns – that depend on epigenetic and environmental variables unfolding during and after differentiation – and diverse embryonic origins (23). While the underlying mechanisms and signaling pathways of fibroblast heterogeneity remain to be fully elucidated, the existence of certain cell-intrinsic properties that are determined during fibroblast development and differentiation is scientifically indisputable (24, 25).

### 2.1 Embryological and developmental origins

The connection between embryological origin and fibroblast subtypes is well known, with each subtype displaying distinct phenotypes and functionalities, as well as colonizing/inhabiting distinct connective tissue compartments and anatomic locations (26, 27). Embryologically, fibroblast-progenitors originate from both mesoderm and neural crest, with the majority of fibroblasts deriving from the presomitic and lateral plate mesoderm (28–30). Interestingly, cell-fate analyses revealed a correlation between the anatomical position of skin fibroblasts and the primitive development: Trunk and limb fibroblasts were linked to the lateral mesoderm, while dorsal and facial fibroblasts could be traced to the dermomyotome in the somite and the neural crest/cephalic mesoderm, respectively (27, 31–33).

As part of their spatial distribution, fibroblasts undergo differentiation and develop positional identities corresponding to their newly found anatomical location – a process that is known to be regulated, at least in parts, by differential expression of multiple homeobox genes (34–36). In the context of these heterogeneous anatomical identities, skin fibroblasts acquire positional memory and individualized functional properties (37). As such, they gain the ability to engage and activate their surrounding microenvironment according to their developmental origin, thus changing the skin appearance and injury outcome (38, 39). Rinkevich et al. demonstrated this capacity in the field of wound healing and revealed that distinct fibroblast lineages from different anatomical origins represent unique cell subtypes with inherent functional diversity in injury repair (4). In their animal study, murine neural crest-derived Wnt1-lineage-positive oral fibroblasts (WPFs) and dorsal Engrailed-1-lineage-positive somitic-derived fibroblasts (EPFs) were transplanted to the dorsal skin and oral cavity, respectively. Strikingly, following transplantation, scar formation on the dorsal skin exhibited characteristics resembling a minimal oral cavity scar phenotype, while typical dorsal scar formation was observed in the oral cavity (4). These findings indicate cell-intrinsic properties of skin fibroblasts – specific to their anatomic position and subtype and independent of microenvironmental niche – that allow the cells to modulate the skin architecture and wound response (27). In order to be able to therapeutically leverage this “seed versus soil” paradigm, future studies focusing on the developmental biology and lineage hierarchies of fibroblasts are essential (18).

### 2.2 Morphological-structural appearances and plasticity

From a morphological perspective, fibroblasts are typically described as large and spindle-shaped cells with flat oval nuclei and sharp processes extending from the ends of the cell bodies. However, it is well documented that fibroblasts are pleiomorphic, and can exhibit various phenotypes, depending on their origin and function (28, 40, 41). Specific to the dermis, fibroblasts in the upper layer tend to be spindle-shaped, whereas fibroblasts in deeper layers are described as more stretched and predominantly stellate with dendritic processes. Both fibroblast subtypes have been reported to maintain their morphology in culture media – an observation that suggests inherent differences between their phenotypes (42, 43). The morphological characteristics of papillary (upper dermis) and reticular (lower dermis) fibroblasts are associated with their functional duties and transcriptional profiles which are described in more detail below (44).

In general, skin fibroblasts exhibit high morphological plasticity which is necessary to fulfill their multi-purpose roles. During the wound healing process, fibroblasts are able to transform into myofibroblasts – a conversion that underlies their fibrotic mechanism and wound contraction/closure ability (45). This healing-related differentiation of fibroblasts is triggered by mechanical signaling, cytokines, and growth factors, with transforming growth factor‐beta (TGF-β) serving as key mediator (46). In response to the TGF-β stimulation, myofibroblasts acquire a network of contractile actin-myosin complexes rich in α-smooth muscle actin (α-SMA). This form of actin is commonly used as a marker for myofibroblast formation (47, 48).

In this context, it is worth mentioning that, contrary to long-held beliefs, it has recently been demonstrated that, upon injury, adipocytes neither convert into fibrogenic myofibroblasts nor contribute to the tissue deposition responsible for scar formation (49). In fact, Gopal et al. challenged the categorical establishment of cellular identities based on the acquisition of cell type-like markers which had previously laid the foundation for the assumption the interchangeability between (myo)fibroblasts and adipocytes (50–52). Using combined data from genetic tracing, single-cell transcriptomics, live imaging/tracking, transplantation assays, and *in vivo* injury models, the researchers were able to substantiate their hypothesis that wound infilitrating adipocytes are clearly distinct from myofibroblasts: while adipocytes were shown to indeed respond to wounding stimuli with subsequent shifts in motility/functionality and to play active supportive roles during the endogenous healing response, they remained lineage-restricted with no evidence of conversion or cell fusion to (myo)fibroblasts (49, 52, 53). Therefore, by demonstrating the irreversible cell fate of mature adipocytes during skin injury, this study contradicts previously assumed reprogramming of adipocytes to fibrosing cells during tissue repair and exemplifies limitations of lineage interplay between stromal cells (49).

In summary, the fibroblast (cross-lineage) hierarchy and its unique plasticity appear more complex than in other cell families and the paradigms of adipocyte-fibroblast trans-differentiation as well as wound-infiltrating myeloid cells as a source of fibroblasts warrant continued research (54). Sinha et al. first described the conversion of blood-borne myeloid to fibroblast-like cells upon tissue injury (55). Specifically, two-thirds of all granulation tissue fibroblasts were found to originate from myeloid cells thought to be primarily wound macrophages. Of note, this phenotypic adaptability was dampened under diabetic conditions (55, 56). Undoubtedly, understanding the plasticity and origin of wound fibroblasts remains pivotal to the development of tissue engineering and cell-based therapeutics, ultimately aiming to manage impaired and chronic wound healing.

### 2.3 Spatial localization and characterization

Dermal fibroblasts are traditionally subdivided synchronously with the histological partition of the dermis into the upper papillary and lower reticular layers (57). While the papillary layer, beneath the epidermis, is marked by a high density of fibroblasts and scattered, thin collagen fibers with large interfibrillar spaces and a high proteoglycan content, the reticular dermis typically features highly organized, thick collagen and elastin bundles and a low cell density (42, 58–61). Moreover, papillary fibroblasts produce higher proportions of collagen VI alpha 1, collagen XVI, decorin, and fibromodulin, contrasting higher levels of collagen I, collagen VI alpha 2, and versican in the reticular ECM (62). In addition, fibroblasts in the papillary layer tend to proliferate, whereas reticular layer fibroblasts respond more sensitively to external cytokines, with their proliferation ability being strongly increased in the presence of TGF-β and Platelet-derived growth factor (PDGF) and with α-SMA expression being detected in a higher proportion of cells (44, 63). The aforementioned variations in fibroblast morphology and function as well as ECM composition and architectural micro-organization triggered the definition of distinct upper and lower layer fibroblast populations with different functional activities, which was subsequently supported by the identification of distinct surface markers for anatomically different lineages (17, 40, 64–66). In recent studies, single-cell sequencing and multiomics analyses confirmed distinct transcriptomic and epigenomic profiles in papillary and reticular fibroblasts while demonstrating that the existence of certain accessible chromatin regions, such as DPP4/CD26, Corin, and Dlk1, is not restricted to specific fibroblast lineages and fluctuates between different functional stages (67, 68). In addition, specialized fibroblasts may localize in the perifollicular area and in skin appendages, such as the dermal papilla, dermal sheath, and arrector pili muscle, implying for a much higher diversity of skin fibroblasts (69).

### 2.4 Gene expression and surface markers

Despite the fact that certain features are shared across fibroblasts, fibroblasts generally exhibit a high degree of heterogeneity in their transcriptional profiles and variability in their gene expression signatures, cell surface markers and differentiation trajectories (49, 70). Accordingly, several attempts have failed to decipher universal fibroblast-surface markers, such as *Platelet-derived growth factor receptors-α (*PDGFRα), Fibroblast-Specific *Protein-1 (*FSP-1), or Vimentin (71–73). Instead, the identification of custom molecular markers for defined subpopulations of skin fibroblasts has become increasingly popular (4, 74). Driskell et al. demonstrated that skin fibroblasts in the murine organism stem from a multipotent progenitor population and differentiate into separate lineages (17). Ultimately, this process was found to give rise to multiple fibroblast subtypes with distinct transcriptional patterns, including papillary, reticular, hypodermal, dermal papilla, and arrector pili muscle fibroblasts. As a result, various lineage-specific molecular markers have been reported, namely CD26/dipeptidyl-peptidase 4 (DPP4) and Leucine-rich alpha-2-glycoprotein 1 (Lrg 1) for papillary fibroblast, delta-like non-canonical notch ligand 1 (Dlk1) for reticular fibroblast and Stem cells antigen-1 (Sca-1) for hypodermal fibroblasts, assorted in an upper papillary and lower pre-adipocyte precursor lineage (17).

In human skin, Tabib et al. used single-cell RNA sequencing to identify two major fibroblast populations via Secreted Frizzled Related Protein 2 (SFRP2)/DPP4 and Flavin containing dimethylaniline monooxygenase 1 (FMO1)/Lymphocyte Specific Protein 1 (LSP1) and five minor populations through the markers Cellular Retinoic Acid Binding Protein 1 (CRABP1), collagen type XI α1 chain (COL11A1), FMO2, Proteoglycan 4 (PRG4), and chromosome 2 open reading frame 40 (C2ORF40) (75). Fibroblast activation protein α (FAP-α) and CD90 have been proposed to differentiate between papillary (FAP^+^CD90^+^) and reticular (FAP^-^CD90^+^) fibroblast subsets in human skin (65). Similarly, Philippeos et al. identified four distinct fibroblast populations in human skin samples and described their molecular markers: while CD90^+^CD39^+^CD26^–^ fibroblasts were detected in the upper dermis, CD90^+^CD36^+^, CD90^+^CD39^+^CD26^+^, and CD90^+^CD39^–^RGS5^–^ fibroblasts were preferentially found in the lower dermis (40). Furthermore, CD26/DPP4, as previously described a marker commonly used to differentiate dermal fibroblasts in mice, was confirmed as a key lineage marker of dermal fibroblasts in human studies. Interestingly, while this cell surface glycoprotein was detectable in a large fraction of the human dermis, its expression was excluded from fibroblasts in the upper dermal layer (40). In a sense, the abundance and incongruence of the identified markers are contrasted with their discriminatory power (76). Proteins that had been hypothesized to differentiate separate lineages may be canonically co-expressed by fibroblasts, thereby forfeiting their effective selectivity.

### 2.5 Functional versatility and responsiveness

The morphological and transcriptional variance of fibroblasts also translates to the functional-operational level: their documented activity is broad, ranging from the synthesis/remodeling of the ECM through the secretion of signaling molecules to the regulation of inflammation and metabolism (28). Ultimately, the destiny of fibroblasts culminates in the creation and orchestration of a tissue-specific foundation for healthy organ viability and, if necessary, for its repair (77).

After identifying the two spatially separated CD26/DPP4^+^ papillary and Dlk1^+^ reticular fibroblasts populations, Driskell et al. also delineated their distinct skin-related functions: namely, during wound healing, migration behavior and ECM deposition differ between the upper and lower dermis-lineage fibroblasts, with the latter targeting excision wounds first, expressing α-SMA as a marker for fibroblast activation. By contrast, fibroblasts from the upper dermis follow to repopulate during re-epithelialization and appear vital for hair follicle formation (17). Furthermore, CD90^+^CD39^+^ fibroblasts located mainly in the upper dermis were found to facilitate the formation of a healthy stratified epidermis in culture and present a more anti-inflammatory phenotype in interferon-gamma (IFN-γ) stimulation assays, whereas lower dermal CD90^+^CD36^+^ fibroblasts showed an increased expression of genes encoding ECM components and inflammatory mediators (40, 78). Philippeos et al. reported an upregulation of the gene expression related to the Wnt/β-catenin signaling pathway in papillary fibroblasts (17, 79). Notably, the current line of evidence points toward a Wnt-mediated and synergistic interplay between basal keratinocytes and papillary dermal fibroblasts that is essential for the maintenance of the fibroblast cellular identity (40). Throughout wound healing, epidermal activation of Wnt/β-catenin has been shown to reprogram adult dermal fibroblasts to a neonatal transcriptional state, leading to fibroblast proliferation and ECM remodeling that originates only from a certain subpopulation of fibroblasts (80). It is important to note that Wnt/β-catenin signaling has also been reported to be mediated and received differently in papillary and reticular fibroblasts, as they react to downstream epidermal Sonic Hedgehog or TGF-β signaling, respectively. As a result, the Wnt-pathway yields divergent cellular responses regarding proliferation, differentiation, and ECM production (81). These findings underscore how the determination of fibroblasts’ cellular identity underlies a combination of both extrinsic signals originating from the spatial setting as well as an intrinsic machinery that includes epigenetic and transcriptional regulatory networks leading to differing responses to external signaling.

More recently, Korosec et al. characterized papillary (FAP^+^CD90^-^) and reticular (FAP^-^CD90^+^) fibroblast subsets which, although not clearly spatially segregated, exhibit functional heterogeneity. Specifically, FAP^+^CD90^-^ fibroblasts featured a superior proliferation potential, while FAP^-^CD90^+^ fibroblasts were able to undergo adipogenic differentiation (65). Analyzing single-cell transcriptomes from more than 5000 skin fibroblasts, Solé-Boldo et al. defined four major dermal fibroblast subpopulations with distinct spatial localizations and functional annotations: secretory-reticular, secretory-papillary, pro-inflammatory, and mesenchymal fibroblasts (64). In contrast, using single-cell RNA sequencing of human skin samples, Vorstandlechner et al. identified six fibroblast clusters with transcriptional and functional heterogeneity. More specifically, they outlined three major fibroblast skin subsets, with one of them being subdivided into four secondary groups (16). Notably, their results challenge the traditional classification of dermal fibroblasts into papillary and reticular fibroblasts, defining subtypes based on transcriptional signatures and corresponding function rather than spatial assignment, as they were unable to identify markers that would allow consistent partition into an upper and lower fibroblast skin lineage. Hence, they functionally characterized fibroblast clusters and found that the most abundant fibroblast subtype almost exclusively expressed DPP4/CD26. This lineage was further established as the main contributor to the production and assembly of ECM components, suggesting a specific special role in wound healing and fibrosis. Of note, this conjecture had been previously documented and could be confirmed in murine and human samples (4, 16, 82). Recently, the Vorstandlechner group analyzed hypertrophic scar tissue and healthy skin, thereby detecting marked differences in the transcriptional signatures of scar fibroblasts (83). Among the highly expressed genes in the scar tissue, a group of serine proteases was particularly prominent. Thus, both DPP4/CD26 and urokinase (PLAU) were further investigated via chemical or genetic inhibition and determined to be majors contributors to the upregulation of matrix protein production and myofibroblast differentiation, with their inhibition leading to a reduction of scarring and an increase in scar quality. While DDP4/CD26 and PLAU could be classified as markers for pro-fibrotic fibroblast subsets in human skin, the researchers also proposed a promising avenue to clinically improve scar quality (83, 84).

A plethora of different fibroblast subgroups with variance regarding transcriptional profiles and functions have hitherto been identified in the skin. Inconsistencies may be due to disparate samples and methodology, thus calling for standardized and transferable research methodology on fibroblasts’ functional heterogeneity in the future (76). **Table 1** provides a detailed breakdown of the currently identified fibroblast subsets in the field of wound healing and skin repair.

**Table 1:** Overview of currently identified fibroblast subsets involved in wound healing and skin repair.

Fibroblast subset	Location	Species	Characteristics	Reference
PDGFRA^+^, CD26^+^, SCA^-^, BLIMP1^-^, LRIG1^+^	Papillary dermis	Mouse	Regenerative, contribution to hair follicle formation	(17)
PDGFRA^+^, DLK1^+^, SCA1^-^	Reticular dermis	Mouse	Profibrotic, wound-induced α-SMA expression	(17)
PDGFRA^+^, Dlk1^+/-^, Sca1^+^	Hypodermal tissue	Mouse	Profibrotic	(17)
PDGFRA^+^, DLK1^-^, LRIG1^+^	Dermis	Mouse	Fibroblast progenitor	(17)
PDGFRA^+^, BLIMP1^+^, DLK1^-^, LRIG1^+^	Dermis	Mouse	Papillary fibroblast progenitor	(17)
PDGFRA^+^, BLIMP1^-^, DLK1^+^	Dermis	Mouse	Reticular fibroblast progenitor	(17)
FAP^+^CD90^-^	Abdominal and breast, papillary dermis	Human	Regenerative, highly proliferative, PDPN, NTN1 expression	(65)
FAP^-^CD90^+^	Abdominal and breast, reticular dermis	Human	Profibrotic, adipogenic, ACTA2, MGP, PPARg, CD36 expression	(65)
CD90^+^CD39^+^CD26^-^	Papillary dermis	Human	Regenerative, anti-inflammatory, support epidermis, expression of papillary markers COL6A5, WNT5A, RSPO1, and LEF1	(40)
CD90^+^CD36^+^	Reticular dermis	Human	Profibrotic, ECM production, include pre^-^adipocytes	(40)
CD90^+^CD39^+^CD26^+^	Reticular dermis	Human		(40)
CD90^+^CD39^-^RGS5^-/+^	Reticular dermis	Human		(40)
SFRP2^+^CD26^+^	Arm	Human	Profibrotic, matrix production and deposition, major subpopulation	(75)
FMO1^+^ LSP1^+^	Arm	Human	Regenerative, inflammatory cell retention, major subpopulation	(75)
COL11A1^+^	Arm	Human	Connective tissue cell differentiation	(75)
CRABP1^+^	Arm, dermal papilla	Human	Regulation of stem cell differentiation in the hair follicle bulge	(75)
SFRP4^+^	Arm	Human	Possible progenitors of PCOLCE2^+^ and/or SFRP2^+^ cells	(75)
PRG4^+^	Arm	Human		(75)
PCOLCE2^+^	Arm	Human		(75)
PDGFRA^+^, CRABP1^+^	Dorsal, upper dermis	Mouse	Profibrotic, Tgfbr2, Tgfbr3 expression	(74)
PDGFRA^+^, CRABP1^-^	Dorsal, lower dermis	Mouse	Regenerative, Tgfbr2, Tgfbr3 expression	(74)
PDGFRA^-^, PDGFRB^+^	Dorsal	Mouse	24% of wound fibroblasts 12 dpw	(74)
EN1 LIN^+^ FIBROBLASTS (“EPFS”)	Dorsal	Mouse	Profibrotic, drivers of fascia matrix migration	(4, 14)
EN1 LIN^-^ FIBROBLASTS (“ENFS”)	Dorsal	Mouse	Regenerative	(4, 14)
WNT1 lin^+^ FIBROBLASTS (“WPFS”)	Oral	Mouse	Regenerative	(4)
PRRX1^+^	Ventral	Mouse	Profibrotic	(15, 85)
PRRX1^-^	Ventral	Mouse	Regenerative	(15, 85)
SCA1^+^, CD34^+^, CD29^+^, EN1 LIN^+^	Dorsal	Mouse	Profibrotic, myofibroblasts and adipocyte precursor cells	(51)
PDGFRA^+^, ADAM12^+^	Ear, perivascular	Mouse	Profibrotic progenitor cells	(86)
CHURC1	Ventral wounds	Mouse	Site-specific expression of developmental genes	(87)
WNT5A	Dorsal wounds	Mouse	Site-specific expression of developmental genes	(87)
MSX1	Cheek wounds	Mouse	Site-specific expression of developmental genes	(87)

To date, a plethora of fibroblast populations in the skin have been isolated, each of which exhibits unique phenotypic and functional characteristics. Different gene expression patterns and cell surface markers allow conclusions to be drawn about their distinct roles in cutaneous wound healing and regeneration. However, high variance in sample origins and disparities in methodology still limit the (inter)comparability, reproducibility, and continuation of previous research findings. Standardized and transferable protocols are, therefore, needed to streamline fibroblast research in the future.

## 3 Skin fibroblasts in wound healing and scarring

If the skin barrier is breached, skin-resident immune and stromal cells cross-communicate and initiate wound healing immediately after the injury (10, 88). Upon skin injury, skin fibroblasts are activated and operate in a phenotypic-variable capacity (89). Current models of wound repair proposed that fibroblasts migrate into the wound area, exhibit contractile properties, and deposit matrix *de novo* onto the granulation tissue prepared by the coagulation cascade. Fibroblasts then trigger the remodeling of this provisional matrix into a mature scar (90). This concept has long held true, however, it has recently been refined as the role of fascia and fascial fibroblasts is being deciphered (2).

In murine back skin, Rinkevich et al. identified two fibroblast lineages that were spatially neighboring but functionally distinct. Fibroblasts expressing the transcription factor Engrailed-1 during their embryological development – termed Engrailed-1 (En1) lineage-positive fibroblasts (EPFs) – exhibited cell-intrinsic scarring properties and contributed to fibrotic processes, such as radiation fibrosis, scar formation in cutaneous wound healing, and cancer stroma growth. By contrast, Engrailed-1-lineage negative fibroblasts (ENFs) promoted skin regeneration and restorative programs, as seen in early embryonal (scarless) wound healing (4). During embryogenesis, the response to wounds shifts from scarless regeneration to the deposition of scar tissue (91, 92). Concurrently, skin-resident ENFs become outnumbered by migratory and proliferating EPFs – a transition that is believed to be causative for the transition in wound healing mechanisms from skin regeneration to fibrotic scarring (14).

In addition, in the ventral dermis, the transcription factor Paired related homeobox 1 (Prrx1) was identified as a marker of a profibrotic fibroblast lineage (15). Prrx1-positive fibroblasts (PPFs) can be regarded as the ventral equivalent to EPFs, accounting for a majority of cellular components as well as ECM deposition in wound repair and demonstrating cell-intrinsic scar-forming properties (15, 85). Prrx1 has been proposed as a pivotal regulator in the activation of fibroblasts to a myofibroblast-like phenotype, thereby highlighting its role in wound closure as well as tumor progression (93).

The Rinkevich group also reported a selective expression of CD26/DPP4 on the surface of 90% of pro-scarring EPFs, thus substantiating the relevance of this marker. Strikingly, the application of diprotin A, an allosteric inhibitor of CD26 peptidase activity, led to significantly decreased scarring and prolonged wound healing, which, in turn, underscored the role of DPP4-positive fibroblasts in tissue deposition (4). Prompted by these findings, Mascharak et al. aimed to investigate the generation of EPFs and their role in postnatal wound repair (94). Using fibroblast transplantation and transgenic mouse models, they identified a dermal ENF subpopulation that generates postnatally derived EPFs by activating En1 expression during wound healing. Interestingly, mechanical tension was found to drive the activation of En1 via canonical mechanotransduction signaling (i.e., Yes-associated Protein (YAP)). Blocking this pathway, either by verteporfin, a YAP inhibitor, or fibroblast-specific YAP knockout prevented the activation of En1 and facilitated skin regeneration by ENFs – with recovery of skin adnexa (glands and hair follicles), ultrastructure, and tensile strength (94). In essence, these insights suggest a dual function of skin fibroblasts in wound healing: a fibrotic, EPF-mediated repair or a regenerative, ENF-mediated regeneration. Accordingly, leveraging fibroblasts’ functional diversity and targeted modulation of fibroblasts may enable scarless wound healing in the future (18, 95). Future studies are needed to translate these findings to humans and, thus, validate its clinical-therapeutic relevance.

## 4 Role of fascial fibroblasts in wound repair

EPFs are not only found in the skin but also in the strata underneath, the fascia (4). The fascial system is a three-dimensional viscoelastic tissue that separates the skin from the body’s rigid structure below. To this end, the fascia consists of tight, mesh-like collagen and elastin fibers, with connective tissue cells, adipocytes, immune cells, and sensory cells forming the cellular foundation (1, 96). While this manifold structure permits the integrity of the microenvironment as well as the regulation of mechanical-metabolic interactions, recent evidence has identified the subcutaneous fascia as an external repository of scar-forming provisional matrix (2, 97).

In fact, Correa-Gallegos et al. demonstrated that deep skin injury triggers fascia mobilization to quickly seal open wounds (2). The skin contraction and scar tissue formation are achieved by the active transport of ECM from the fascia, deep beneath the wound edges. More specifically, after a wound develops, fascial EPFs rise to the skin, piloting their local composite matrix (including embedded vasculature, nerves, and immune cells) into the wound to form – in coordination with the coagulation cascade – the provisional wound matrix (referred to as patch repair) (97). Of note, the genetic ablation of these fascial fibroblasts hindered the matrix from skin-homing and, subsequently, led to defective scars. Similarly, the placement of an impermeable film barrier beneath the skin prevented fibroblasts from migrating into the upper dermal layers and resulted in chronic open wounds that fail to heal. In sum, this research proposed a revised version of the wound repair paradigm following deep skin injuries, with a ‘scar primordium’ being steered by sentry fibroblasts instead of *de novo* matrix wound deposition through dermal fibroblasts (2). The highly specialized fibroblasts are harbored in a prefabricated matrix of the fascia that homes into the wound area as a movable sealant, thereby dragging along blood vessels, peripheral nerves, and macrophages. Interestingly, the effect of dermal and fascial EPFs on scarring was found to differ. While the quantity of fascial EPFs was significantly associated with wound size and scar severity, no such correlation could be established for their dermal counterparts. Accordingly, in deep wounds with increased scar size, the number of fascial EPFs doubled that of dermal EPFs. By contrast, in superficial wounds, the abundance of fascial EPFs was substantially reduced, and so was the scar severity. It is, therefore, reasonable to investigate the fascia as the root of excessive scarring via wound native cells such as pro-scarring EPFs (2, 98).

More recently, underlying mechanisms behind the fascia cell mobilization following deep skin injuries have been deciphered. In their full-thickness murine wound models, Jiang et al. revealed a collective migration pattern of fascial EPFs that mounted in a crescendo, with distinct EPF foci coalescing into large collective swarms that moved toward wound centers (5). It is worth noting that this swarming-like cell migration during scar formation was found to occur exclusively in fascia fibroblasts. In the oral mucosa, an anatomic site lacking fascia and with minimal scarring, fibroblasts showed different cell trajectories/dynamics and no swarming activity upon wounding. Furthermore, during wound repair and scarring, fascial EPFs upregulate the expression of N-cadherin, a calcium-dependent cell-cell adhesion molecule N-cadherin. The selective inhibition of N-cadherin hindered EPFFs’ swarming-like aggregation, thereby reducing skin contraction and resulting in decreased scarring. These findings point toward a crucial role of N-cadherin in facilitating fascial cell mobilization with subsequent scarring (5). As the mechanism of N-cadherin-mediated fibroblast aggregation has also been confirmed in human tissue, N-cadherin may serve as a clinically relevant lever to curb fascia mobilization and, thus, excessive scar formation (99).

Along with N-cadherin, cell-cell communication plays an integral role during the patch-like repair of deep skin wounds. In their murine full-thickness wound models, Wan et al. identified gap junctions, more precisely connexin43 (Cx43, a structural protein as a component of gap junctions), as the molecular key for fascial matrix movement and associated scar formation (100). Indeed, the expression of Cx43 in response to wounding was found to be markedly upregulated in fascial EPFs, the fibrogenic fibroblast lineage responsible for scar formation following deep skin injuries. Live imaging of fascia fibroblasts and fate tracing of the fascia extracellular matrix revealed that the inhibition of Cx43 interferes with calcium signal oscillations in cultured fibroblasts – which impaired the collective migration of fascial EPFs necessary to mobilize the fascia matrix. As an *in vivo* morphological result of these mechanistic interactions after Cx43 blockade, the researchers observed a reduced scar formation with less collagen content and a lower expression of the fibrogenic cell marker CD26/DPP4. In other words, mammalian scarring in response to deep wounds involves selective gap junction expression and cell-cell communication via Cx43 for the upshot of fascial cells and matrix into the skin wound area. Therefore, through a clinical-translational lens, targeting Cx43 or calcium signaling underlying the scarring response may provide therapeutic avenues to curtail fibrosis and scarring (100).

In this context, it is important to highlight that both Cx43 and N-cadherin share cell adhesion functions and both may direct migrations of cell populations. Yet, to date, the signal cascade between these mediators remains incompletely understood. While the hypothesis that Cx43 operates as a transcription factor upstream of N-cadherin seems plausible, future studies are needed to delineate the molecular-cellular interplay in depth (101).

In 2022, Rajendran et al. identified another player that contributes to fascia mobilization during deep wound healing (102). Namely, p120 catenin was highly expressed in EPFs throughout the wound repair process, regulating the supracellular organization required for fascia mobilization toward skin wounds. Accordingly, silencing of p120 catenin via adeno-associated virus serotype 8 (AAV-8)-mediated short hairpin RNA (shRNA) *in vivo* blocked fascia fibroblasts’ collective directional migration, thereby yielding improved wound repair in form of significantly reduced scars. These insights are relevant from two perspectives: (i) p120 catenin is an adhesion junction protein that can bind directly to N-cadherin which is also known as an essential mediator for fascia mobilization. It is, therefore, intriguing to speculate that the two molecules interact, with p120 catenin presumably either upregulating or stabilizing N-cadherin. The identification of p120 catenin as an orchestrator of the EPFs’ supracellular organization plus its likely interplay with N-cadherin marks another step toward unraveling the biochemistry behind fascia mobilization. (ii) AAVs may represent effective delivery vehicles for therapeutic modulation of the signaling cascade underlying the patch-like repair after skin wounding (102).

**Figure 2:**
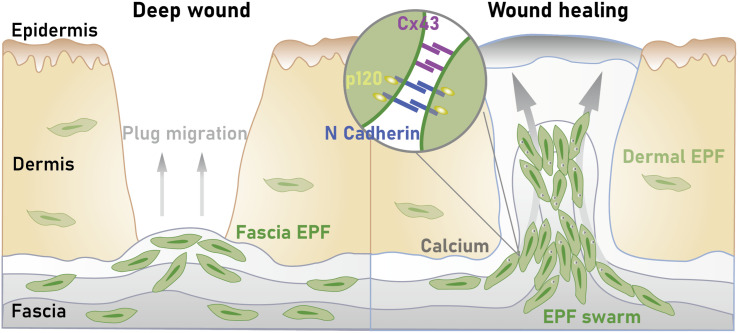
Current conception of patch-like scar repair after deep skin wounds. Fascial engrailed-positive fibroblasts (EPFs) drag local composite matrix into the wound, with a collective migration pattern of EPFs underlying this conveyor belt-like steering. During this fascia mobilization, cell-cell adhesion and communication via N-cadherin and connexin43 (Cx43) play an integral role, facilitating swarm-like aggregation and ultimately sealing of the open wounds with dense plugs of pre-fabricated fascial matrix. Over time, this provisional fascia-derived skin barrier is remodeled into a mature (excessive) scar.

Taken together, the current line of evidence points to a dual mechanism of wound healing: while superficial injuries typically trigger the migration of dermal fibroblasts into wounds, where they locally deposit wound matrix *de novo* onto granulation tissue delivered by the coagulation cascade, deep skin wounds initiate different molecular machinery with the fascia as the anatomical epicenter. Instead of cutaneous regeneration, large open skin surfaces are physically sealed with dense plugs of connective tissue matrix known as scars. Recent findings suggest that such scarring may be the result of fascial EPFs collectively migrating toward the wound area, whilst dragging their surrounding prefabricated ECM matrix with them (**Figure 2**). Thus, during this patch-like repair, the fascial matrix may serve as a provisional barrier that is remodeled over time into a mature scar. The new understanding regarding the relevance of fascia and its resident fibroblasts throughout wound healing routines ought to be taken as an incentive to investigate the (reciprocal) interaction of this tissue and its cells with the skin. In fact, unraveling the cellular skin-fascia dynamics might be the key to forestalling excessive scar formation after deep injury and achieving aesthetically superior skin repair.

## 5 Future directions and clinical-translational perspectives in wound healing and skin regeneration

Scars replace the healthy reticular connective tissue substructure with an irregular dense meshwork of matrix fibers, thereby reducing its biomechanical and physiological functionality. In the Western hemisphere, an estimated number of 100 million patients develop scars each year (103). As such, scarring – in particular, excessive scars including hypertrophic and keloid scars – represent a pressing clinical-medical challenge and a significant economic burden to the global healthcare system (104). It is worth mentioning that in the U.S. alone, the annual cost of treating skin scarring amounts to more than 10 billion dollars (105).

### 5.1 Targeting of fibroblast impact during fascia mobilization

Traditionally, scarring has been conceptualized as a pathology of *de novo* matrix synthesis and deposition by fibroblasts at the wound site. Based on this prevailing understanding, anti-scar and anti-fibrotic treatment modalities have been developed. Yet, while a broad armamentarium of therapeutic options is available, the efficacy and/or safety of current scar management remain limited. In addition, to date, the underlying pathomechanisms are only fragmentarily understood and offer poor targeting options. As a consequence, clinical translation is lagging, while scar patients seek therapeutic remedies (18, 98).

This stagnation is contrasted by the emerging evidence on the etiology of skin scars from prefabricated matrix in the subcutaneous fascia which holds the potential to revolutionize anti-scar care. In fact, independent of *de novo* matrix deposition, targeting the fascia mobilization prior to patch-like repair may prevent excessive scarring (97). In this context, two therapeutic approaches (with varying foci) appear to be particularly promising in the short and long term:

(i) In response to injury, the expression of N-cadherin adherens-junctions and Cx43 gap-junctions were upregulated specifically in fascial EPFs (5, 100, 102). The adhesion and communication between fascial EPFs through these junctional structures set the stage for collective cell migration, thereby steering the fascial matrix toward the wound center (5). This conveyor belt-like function of fascial EPFs and the movement of the subcutaneous fascia was abrogated by the therapeutic blockade of Cx43 via the subcutaneous injection of GAP 27, a Cx43 mimetic and inhibiting peptide. As a result, the full-thickness wound sizes of GAP 27-treated mice were significantly reduced in comparison to control wounds, with the amount of collagen I as well as the number of fascial EPFs and F4/80^+^ macrophages being decreased (100). Similarly, Exherin (a selective inhibitor of N-cadherin) treatment hindered the aggregation of EPFs and caused them to disperse randomly in the fascia – in contrast to swarms and centripetal migration patterns seen in controls. Accordingly, upon chemical inhibition of N-cadherin, fascial EPFs’ swarming and subsequent fascia mobilization into the wounds was absent which ultimately resulted in minimal scar formation and distinct collagen fiber architecture (5). Of note, cell adhesion is known to be a calcium-dependent process. Therefore, it is not surprising that the administration of the calcium (Ca2^+^) chelator ethylene glycol tetraacetic acid (EGTA) or Nifedipine, a Ca2^+^ channel blocker, resulted in significantly reduced scarring and lack of collective cell behavior, respectively (100). These findings can be understood as a testament to the general value of pharmaceuticals inhibiting fascia mobilization, with high clinical anti-scarring potential (106–108).

While these synthetic chemical inhibitors have shown promise in preclinical studies, translation into clinical applications faces a series of challenges. Differences between murine and human fascia anatomy as well as the heterogeneity of fascial fibroblast subsets limit the transferability and generalizability of the results obtained (**Table 1**). Preclinical models may indeed serve as valuable tools for initial assessments, yet, prior to clinical implementation, the efficacy and safety of these inhibitors need to be thoroughly evaluated through well-designed clinical trials with human patients (109, 110). In addition, potential targets, such as N-cadherin, Cx43, and Ca2^+^, play crucial roles in a variety of physiological processes across different tissues and organs (111–113). Inhibition of these molecules for anti-scar therapy carries the risk of off-target effects, which could lead to unintended complications. It is, therefore, essential to develop precise drug delivery methods that specifically target the skin fascia and minimize the impact elsewhere. Despite these challenges, the encouraging insights provided by the preclinical data warrant future investigation. Advances in drug delivery technologies, such as innovative nanoparticles or localized delivery systems, may help circumvent the pitfalls and drawbacks associated with the systemic administration of synthetic chemical inhibitors (114, 115).

(ii) Another approach to disrupt the pathway underlying patch-like repair is viral-based genetic modification, aiming to regulate key molecules. Jiang et al. documented the successful administration of adeno-associated virus serotype 6 (AAV-6) viral particles expressing Cre recombinase into the fascia around wounds in N-cadherin floxed mice (5). As a result of this injection, the transduced Cre-expressing fascial fibroblasts near the wounds lost N-cadherin, with the Cre-expressing virus resulting in a 65% decrease of the N-cadherin expression in the scar environment. Strikingly, N-cadherin patchy knockout led to a significantly modified scar architecture (namely, less complex and more porous lattice), suggesting that N-cadherin loss may be associated with improved wound quality (5).

The authors also proposed an independent strategy to locally knout out N-cadherin using CRISPR-Cas9. To this end, AAV6 viral particles with guide RNA targeting murine N-cadherin exon 1 were injected into the fascia around wounds of Cas9-expressing mice. The downregulation of N-cadherin was found to result in smaller scars *in vivo*. In addition, in order to generate offspring in which only fascial EPFs express Cas9, En1-Cre mice were crossed with Cas9 knock-in mice. The local knockout of N-cadherin in EPFs yielded similar results, i.e., reduced scar sizes and disrupted EPF swarming (5).

The potential of AAV vectors in modulating endogenous wound responses was also demonstrated by Rajendran et al. (102) After screening different AAV serotypes, AAV-8 was found to exhibit the highest transduction efficacy in fascial fibroblasts. In addition, the authors documented both the methodology and effect of AAV-8-mediated shRNA silencing of p120 *in vivo*. As mentioned above, knocking down the molecular trigger p120 in fascial fibroblasts via this AAV-8 system reduced ECM mobilization, lowered collagen expression, and significantly decreased scar size. The subcutaneous delivery of AAV-8 p120 shRNA effectively hindered the collective migration of fascial fibroblasts required for fascia mobilization toward the wound center. Accordingly, the wounds of AAV-8 p120 shRNA-injected mice were markedly smaller. It is important to emphasize that multiple doses of AAV-8-mediated p120 knockdown are needed to maintain the silencing effect (102). Despite the undeniable potential of viral vector-based gene therapy, persistent hurdles hamper its clinical-translational applicability. To date, our understanding of its therapeutic efficacy is largely based on animal models, with the responses of the human organism’s innate and adaptive immune system against viral vectors remaining ill-defined. This knowledge gap is widened by high-cost manufacturing and production challenges of modified viruses as delivery vehicles (116). Still, these findings of effective gene modification in the skin and fascia call for further pre-clinical investigation whilst paving the way for new routes in the therapeutic management of scar and wound pathologies.

### 5.2 Mechanotransduction and wound fibroblasts

**Figure 3:**
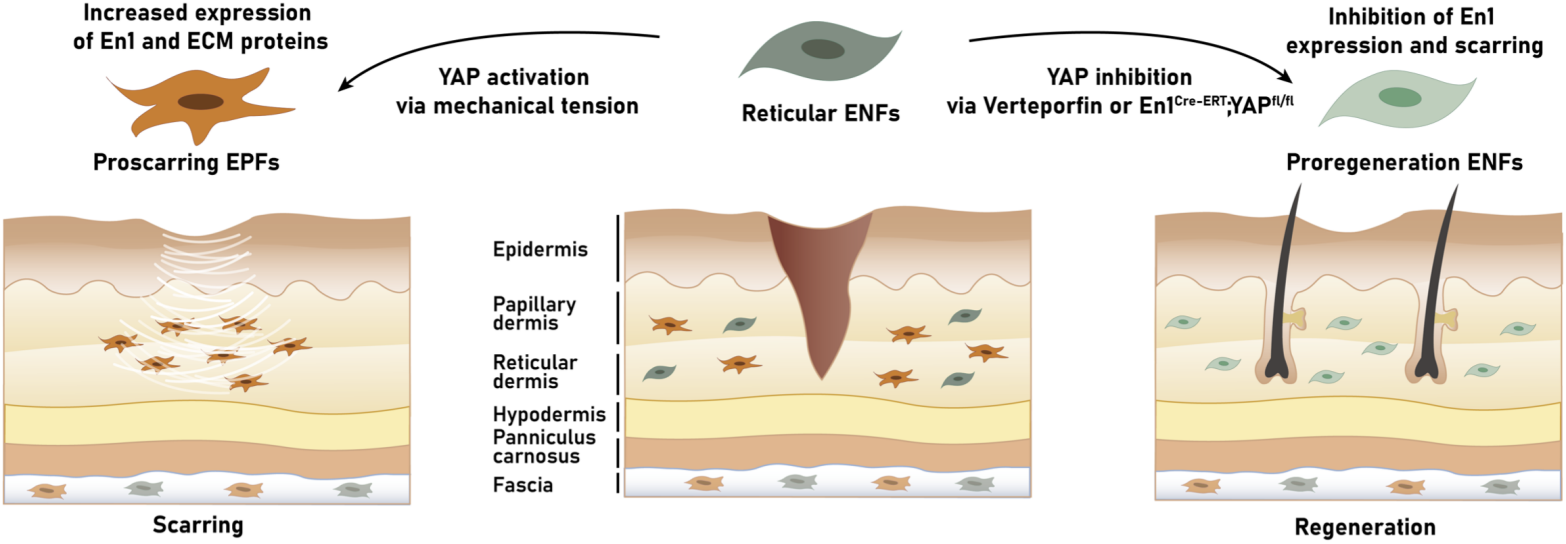
Mechanotransduction-based plasticity of engrailed-1 negative fibroblasts (ENFs) at the crossroads between scarring and regeneration. Mechanical tension applied to skin wounds may trigger Yes-associated protein (YAP) mechanotransductive signaling in ENFs and (re)activate Engrailed-1 (En1), thereby giving rise to proscarring En1 positive fibroblasts (EPFs). As a result of this cellular conversion from ENFs to EPFs, skin injuries are sealed with scar matrix following fibrotic wound healing. In contrast, blocking mechanotransduction signaling – with either verteporfin, a YAP inhibitor, or fibroblast-specific transgenic YAP knockout – prevents En1 activation and leads to wound regeneration, with ENFs regrowing skin appendages (such as hair follicles and glands), restoring mechanical strength, and reestablishing healthy basket-weave matrix ultrastructure.

Originally, the pro-scarring Engrailed-1 (En1) lineage-positive fibroblasts (EPFs) were postulated to be the progeny of fibroblasts that expressed En1 exclusively during early embryonic development. In contrast, En1 lineage-negative fibroblasts (ENFs) were considered as a separate lineage of skin fibroblasts that would not share the history of En1 expression and mainly contribute to the formation of the dermis instead of fibrotic processes during wound healing (4, 117, 118). However, more recently, Mascharak et al. provided evidence of the inherent plasticity of ENFs (94). More specifically, in their murine studies, the researchers demonstrated that within adult wounds En1 expression can be reactivated postnatally in ENFs. After such phenotypic conversion, the fibroblasts are referred to as postnatal EPFs (pEPFs) [in distinction to EPFs with an embryonic expression of En1 (eEPFs)], with pEPFs generating up to 50% of all wound fibroblasts. Interestingly, the ENF-to-pEPF transition was found to depend on mechanical cues: while the phenomenon of En1 reactivation could not be observed in reticular ENFs cultured on soft hydrogels, an upregulation of En1 and EPF-transition was seen in those cultured on high-stiffness tissue plastic. In addition, the pharmacological inhibition of Yes-associated protein (YAP), the key effector of the mechanotransductive pathway, via verteporfin as well as diphtheria toxin-mediated targeted ablation of pEPFs blocked the activation of En1 and promoted ENF-mediated skin regeneration with restoration of functional sebaceous glands and hair follicles. Therefore, this study yielded two groundbreaking findings (**Figure 3**): first, the notion of definitive segregation of ENF and EPF with distinct lineage-specific functions was honed. Second, the achievable blockade of the conversion between a non-scarring to a scarring stromal lineage (ENFs-to-EPFs) via manipulation of the mechanoresponsive signaling may allow for scarless wound repair (94). Nevertheless, the study also raised a series of questions that need to be addressed in the future, such as whether YAP is a critical upstream regulator of ENF-to-pEPF conversion or of EPFs, or the unidentified routes by which En1 transcriptional activation turns on matrix remodeling functions in wound fibroblasts.

Chen et al. have analyzed the relationship between mechanotransductive signaling and fibroblast fate in a porcine model of split-thickness skin grafts (119). Notably, the blockade of mechanotransduction (via small-molecule focal adhesion kinase inhibitor [FAKI] embedded in a hydrogel) mitigated fibrosis in skin grafts by reducing contracture, restoring collagen architecture, and improving biomechanical properties. When investigating the cellular-molecular mechanisms behind these micro- and macroscopic observations, the researchers observed that the disruption of mechanotransduction at early stages had a greater effect on myeloid cells rather than fibroblasts: specifically, the pharmacological blockade of mechanotransduction promoted myeloid CXCL10-mediated anti-inflammatory transcriptional profiles while the transcriptional activity of fibroblasts remained at a relatively stable level. Controlling mechanical signaling in myeloid-lineage cells during early time points modulated the expression of inflammatory signals with a direct impact on fibroblasts’ phenotypes. At later phases of wound repair, mechanical forces pushed fibroblasts toward profibrotic differentiation fates, with FAKI hydrogel administration modulating mesenchymal fibroblast differentiation states to block such cascading and shifting fibroblast toward proregenerative, adipogenic (lipofibroblast) states similar to unwounded skin. It is important to note that these findings of different fibroblast transcriptional trajectories could also be confirmed in patient skin and scar samples, thereby demonstrating the human equivalence and relevance. In conclusion, Chen et al. indicated a complex crossplay between immune cells and fibroblasts during different stages of wound repair that warrants further investigation. In addition, they established that mechanical stress causes fibroblasts to follow a distinct profibrotic program that can be pharmacologically inhibited and driven toward a pro-regenerative commitment via FAKI – with improved healing outcomes through early anti-inflammation and late regeneration. Future research is needed to determine the generalizability of these findings from a split-skin graft wound model and analyze the involvement of other cell types at the single-cell level (119).

**Figure 4:**
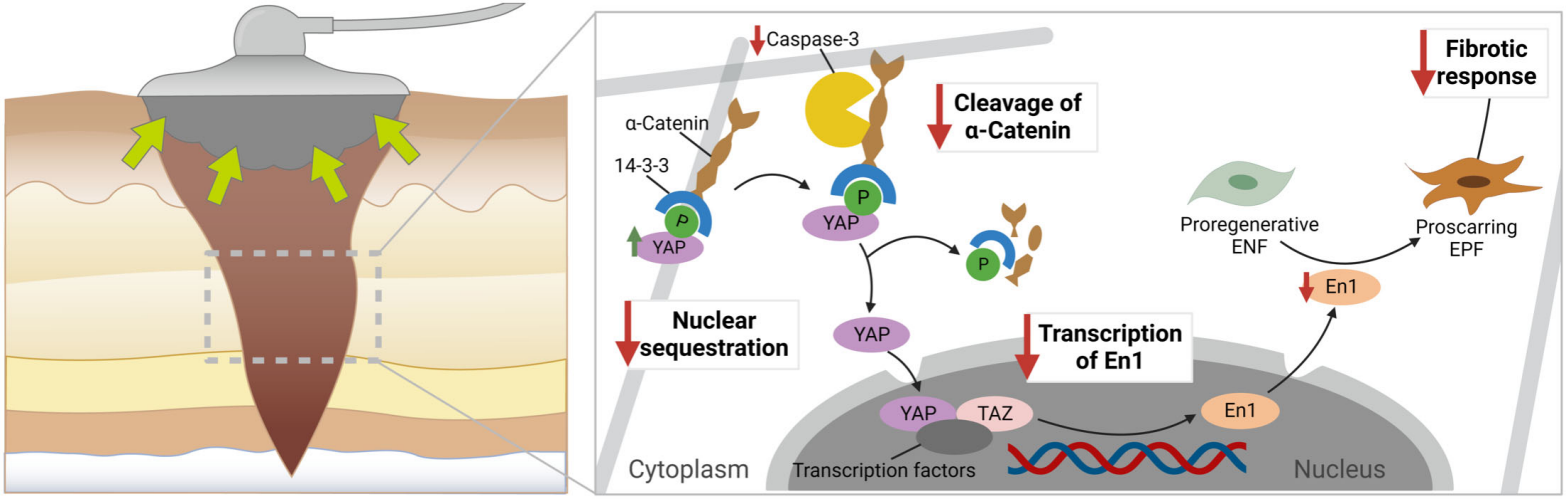
Proposed cellular-molecular mechanisms in negative pressure wound therapy (NPWT) during wound repair. It is hypothesized that mechanotransduction (in the form of increased tension between the cell and the extracellular matrix) results in an upregulation of the mechanotransducer Yes-associated protein (YAP). In the cytoplasm, under healthy conditions, YAP is bound to a-catenin via 14-3-3, which hinders its nuclear sequestration. Yet, in fibrotic processes, caspase-3 is upregulated, leading to the cleavage of a-catenin and translocation of YAP to the nucleus. Downstream, YAP can promote the transcription of En1 in the nucleus. The increased expression of En1 is then paralleled by an increased generation of proscarring En-1 positive fibroblasts. The therapeutic application of NPWT significantly modifies this signaling route, thus resulting in a decoupling of YAP and En1: while NPWT induces an increase in YAP via mediation of mechanotransductive cues, it, strikingly, also leads to a downregulation of En1. By decreasing the expression of caspase-3, the cleavage of a-catenin is reduced following NPWT application, thereby preventing the nuclear sequestration of YAP. As a result, the transcription of En1 is decreased, which translates into diminished numbers of proscarring EPFs. In other words: NPWT (fore)stalls the fibrotic wound response and thwarts excessive scar formation.

A seeming caveat of the triggering effect by biomechanical stimuli on the activation of En1 in ENFs (i.e., promotion of profibrotic/-scarring cellular programs) lies in the well-documented anti-scarring effect of negative pressure wound therapy (NPWT) which is based on the concept of mechanotransduction and micromechanical stimuli. NPWT, also referred to as vacuum-assisted wound closure, is a widely accepted therapeutic modality for the management of acute and chronic wounds. In this wound dressing system, an open-cell foam or gauze is placed in the wound cavity and a controlled negative pressure is applied to remove excess exudate and cellular debris from the wound bed (120). Its efficacy (i.e., the promotion of wound healing) is well-documented and mainly attributable to four mechanisms of action, namely (i) microdeformation, (ii) macrodeformation of the tissues, (iii) drainage of extracellular inflammatory fluids, and (iv) stabilization of the wound environment (121, 122). Wu et al. investigated the molecular ramifications of NPWT in the scarring pathways using a murine diabetic wound model (123). While NPWT was found to be associated with increased YAP, its application also resulted in a significant decrease in EPFs. Further analyses confirmed this ambivalent effect of NPWT: markers from the fibrotic cycle pre-YAP sequestration (namely, fibronectin and RhoA) were upregulated, whereas downstream factors of YAP sequestration, such as En1, Heat shock protein 47 (a chaperone necessary for the maturation of pro-collagen to collagen), and collagen deposition were decreased after the application of NPWT. With these results in mind, Wu et al. proposed a lack of YAP nuclear sequestration as the plausible mechanism underlying the observed cellular-molecular shifts, with NPWT decoupling YAP from En1 activation and thus improving the final scar appearance (**Figure 4**) (123).

Theoretically, the modification of the scar response after skin wounding by manipulating mechanotransduction signaling through small-molecule pharmacological inhibition, NPWT, or genetic deletion holds the potential to revolutionize wound and scar care. Yet, future studies are needed to decipher the exact interplay between biomechanics and wound healing (124). Answering the research question of whether EPFs and ENFs exist in human skin (or at least equivalents) remains a conditio-sine-qua-non prior to any clinical translation of En1-related anti-scar therapies.

### 5.3 Pedigree studies of wound fibroblasts

Recently, Rinkevich’s group has revealed the root of cellular pathways underlying the wound healing cascade (125). In fact, the researchers identified multipotent fibroblast progenitors marked by CD201 expression in the subcutaneous fascia. In a finely and spatiotemporally balanced sequence, this CD201^+^ cell lineage was shown to give rise to all specialized wound fibroblast subtypes required for the progression of wound repair. More specifically, during the initial transition of these CD201^+^ fascia progenitors into proinflammatory fibroblasts, retinoic acid sustained inflammation whilst preventing the conversion into myofibroblasts. In other words: retinoic acid represented a fibroblast differentiation checkpoint, regulating the entry from the fascia progenitor into the pro-inflammatory state (125).

The subsequent acquisition of the (proto-)myofibroblast state had been previously characterized in culture, with mechanical stress (through YAP-TAZ-signaling) being documented as one trigger factor. Yet, while the general relevance of mechanotransduction could be verified, the Rinkevich group highlighted the key role of hypoxia signaling via hypoxia-inducible factor 1-α (Hif1-α). The chemical inhibition of Hif1-α activity augmented the abundance of proinflammatory fibroblasts, whereas α-SMA+ myofibroblast differentiation and tissue contraction were blocked *ex vivo* – with analogous observations (i.e., delayed wound closure) *in vivo*. Thus, Hif1-α was found to control the proinflammatory exit and orchestrate the appearance of (proto-)myofibroblasts. In this context, it is also worth noting that both checkpoint signals (retinoic acid and Hif1-α) chronologically and functionally preceded known (proto-)myofibroblasts inducers such as TGF-ß and YAP-TAZ (125).

These insights of early signals underlying the graduated generation of phenotypically distinct wound fibroblast types call for clinical-therapeutic translation. Targeting both the retinoic acid and Hif1-α signaling routes along the differentiation trajectory may offer novel strategies to effectively modulate fibroblast states (126). Accordingly, fine-tuning of myofibroblast activity by accurately manipulating the gatekeeping function of Hif1-α could prevent excessive scar formation/contraction without compromising the necessary proinflammatory wound state. The fact that CD201+ fibroblast differentiation from naïve fascia progenitor cells through the inflammatory state to myofibroblasts has also been conserved in human keloid and psoriatic bioinformatics datasets renders clinical-practical leveraging of these basic science findings in the management of impaired wound healing and skin diseases all the more auspicious (125).

### 5.4 Sequencing methods and wound fibroblasts

Single-cell RNA-sequencing (scRNA-seq) technology represents the state-of-the-art approach for investigating the heterogeneity and complexity of RNA transcripts within individual cells and elucidating the cellular complexity in high detail (127, 128). Yet, persisting hurdles limit the scientific value of current sequencing methods. Such obstacles include low capture efficiency, distinct dropout rates, static snapshots of cellular states, and loss of spatial information (11, 129).

The combination of low capture efficiency and pronounced dropout rates leads to increased data noise and variability, ultimately complicating the computational scRNA-seq analysis and clustering (130). Of note, the clustering per se has been perplexing the scientific community since the advent of scRNA-seq in 2009. For instance, Tabib et al. analyzed six skin biopsy samples by scRNA-seq and found two major fibroblast populations defined by distinct genes such as SFRP2 and FMO1. The authors further reported five minor fibroblast populations with discrete gene expression (e.g., CRABP1 or COL11A1) (75). He et al. performed scRNA-seq five patients with atopic dermatitis and revealed four different fibroblast populations (i.e., COL11A1^+^LAMC3^+^, APOE^+^ABCA6^+^, COL6A5^+^COL18A1^+^, FBN1^+^MFAP5^+^) (131). Overall, future studies are warranted to improve capture efficiency, reduce dropout rates, and determine a uniform gold standard for cluster configuration.

Moreover, the dynamic process of wound healing remains elusive for current sequencing approaches, as such techniques only analyze the cellular status quo (132). To provide a broader view of the cell fate rather than the cell state, Trapnell et al. used single-cell RNA-Seq data collected at multiple time points and programmed an unsupervised algorithm (Monocle) capable of time-series gene expression analyses (133). Guerrero-Juarez et al. deployed Monocle to group wound fibroblasts into twelve clusters. Thus, the authors could demonstrate that wounding drives fibroblast heterogeneity (74). While the concept is intriguing, further research is needed to refine this method and address persisting obstacles. For example, Monocle’s computational tree is programmed to connect individual cells resulting in a complex tree architecture. In such cases, tree inference is also associated with highly variable results (134).

Further, scRNA-seq analyses are still limited to molecular and cellular information without integrating the specific skin layers and structures (135, 136). To investigate the spatial organization of skin, co-detection by indexing (CODEX) uses DNA-conjugated antibodies and complementary fluorescently labeled DNA probes to visualize up to 60 cellular markers in situ (137, 138). Alternatively, the visium spatial transcriptomics platform is based on slide-bound, single-stranded DNA probes to capture polyadenylated mRNA (139). Foster et al. deployed this technique to shed light on the spatial transcriptomic profile of mouse wounds. The authors found that the distinct skin layers epidermal, indeed clustered based on anatomically specific transcriptional profiles. For instance, they reported that Pdgfra was expressed by fibroblasts within the dermis, while Krt6b expression marked epidermal keratinocytes (67). Overall, spatially resolved transcriptomics might have broad implications for the study of wound fibroblasts in tissue repair by combining the positional context with molecular and cellular data.

## 6 Conclusions

Skin injury drives a temporally- and spatially-synchronized cascade of biological processes, aiming to restore cutaneous integrity whilst minimizing tissue damage. During this highly complex stromal-immune interplay, fibroblasts hold a key role. In fact, their field of activity is multifaceted and ranges from the deposition of wound matrix through serving as a cellular conveyor belt for steering fascia connective tissue into the wound niche with subsequent dense scar plugs. In order to achieve such functional heterogeneity, distinct fibroblasts operate in specialized ways within the skin environment. Yet, this choreography of wound healing processes by fibroblasts appear double-edged: while the cells are vital for healthy wound repair, they may also contribute to fibrosis and excessive scarring, or alternatively impede wound healing from progressing such as occurs in diabetic and ulcerative wounds. Therefore, a comprehensive understanding of fibroblast diversity and versatility can help both decipher wound pathologies and effectively treat skin injuries with the penultimate goal of achieving scarless wound repair.

## Author contributions

Conceptualization, SK, H-GM, DJ, and YR; Methodology, SK, H-GM, DJ, and YR; Investigation, SK, SB, RG, RD, LK, MK-N, FD, and BP; Writing—original draft, SK and SB; Writing—review & editing, LK, MK-N, FD, BP, H-GM, DJ, and YR; Visualization, SK, RG, RD, and DJ; Supervision, BP, H-GM, DJ, and YR. All authors have read and agreed to the published version of the manuscript.
